# Transcriptome analysis reveals brown adipogenic reprogramming in chemical compound-induced brown adipocytes converted from human dermal fibroblasts

**DOI:** 10.1038/s41598-021-84611-0

**Published:** 2021-03-03

**Authors:** Yukimasa Takeda, Toshikazu Yoshikawa, Ping Dai

**Affiliations:** 1 Department of Cellular Regenerative Medicine, Graduate School of Medical Science, Kyoto Prefectural University of Medicine, 465 Kajii-cho, Kawaramachi-Hirokoji, Kamigyo-ku, Kyoto, 602-8566 Japan; 2 Louis Pasteur Center for Medical Research, 103-5 Tanaka-Monzen-cho, Sakyo-ku, Kyoto, 606-8225 Japan

**Keywords:** Cell biology, Molecular biology

## Abstract

Brown adipogenesis contributes to controlling systemic energy balance by enhancing glucose and lipid consumptions. We have previously reported chemical compound-induced brown adipocytes (ciBAs) directly converted from human dermal fibroblasts using a serum-free medium. In this study, genome-wide transcriptional analysis was performed in ciBAs in comparison with the control fibroblasts. A broad range of integrated gene expression was enhanced in functional groups including tricarboxylic acid cycle, electron transfer chain, triglycerides metabolism, fatty acid and glucose metabolism, and adaptive thermogenesis. The results suggested that the chemical conversion underwent metabolic and mitochondrial reprogramming closely associated with functions in brown/beige adipocytes. Moreover, we also compared the transcriptional changes to those of adipocyte browning in adipose tissue-derived mesenchymal stem cells (AdMSCs). Transcriptome analysis indicated that the same sets of metabolic and mitochondria-related genes were similarly changed in the adipocyte browning. Interestingly, ciBAs more expressed *Ucp1*, while AdMSC-derived adipocytes predominantly expressed *Ucp2*. UCP1 protein was also more expressed in ciBAs than in AdMSC-derived adipocytes. Based on the evidence that UCP1, but not UCP2, is responsible for adrenergic thermogenesis, ciBAs could be a promising model for human beige adipocytes applicable for basic research, drug development, and clinical uses.

## Introduction

Adipose tissues regulate energy homeostasis by two types of adipocytes in response to nutritional states and environmental temperature changes^1,2^. White adipocytes have a unilocular lipid droplet to store lipid as an energy source during starvation and fasting. In contrast, brown adipocytes possess more mitochondria and multilocular lipid droplets to actively consume fatty acids for heat production to maintain body temperature. Rodents have two types of thermogenic adipocytes, classical and beige adipocytes^3^. Accumulated evidence has suggested that mouse beige adipocytes are initially differentiated from specific progenitor cells in inguinal white adipose tissues by either chronic cold acclimation or a long-term treatment with β-adrenergic receptor agonists^4^. Inducible beige adipocytes are likely interconverted between dormant and active states to control thermogenic activity and whole-body energy metabolism^5^. Brown adipocytes in adult human are present as beige adipocytes dispersed within multiple subcutaneous adipose depots such as supraclavicular and paravertebral regions^6,7^. Human brown adipocytes more resemble mouse beige adipocytes rather than classical brown adipocytes in terms of gene expression and the transient states of a thermogenic capacity dependent on uncoupling protein 1 (*Ucp1*)^8,9^. Recent studies have shown that mouse beige adipocytes contribute to metabolic pathways such as glucose tolerance, insulin sensitivity, white adipose tissue fibrosis, and hepatic steatosis^10–12^. Therefore, human beige adipocytes might have therapeutic potential to improve systemic glucose and lipid metabolism, which is associated with prevention of obesity and related metabolic diseases.

Mesenchymal stem cells (MSCs) isolated from various tissues have a potential to differentiate into chondrocytes, osteocytes, and adipocytes^13^. Emerging evidence suggested that MSCs can be differentiated into both white- and brown-like adipocytes under proper culture condtions^14,15^. Single-cell transcriptome sequencing implicates that adipose tissue-derived MSC (AdMSC) possess several subtype populations differentially associated with adipocyte functions such as lipid storage, adipokine secretion, mitochondrial biogenesis, and thermogenic capacity^16^. One of the subtypes was more responsive to the treatment with Forskolin and induced the expression of *Ucp1*. The induction of cellular cAMP by Forskolin is associated with *Ucp1* transcription through Protein kinase A (PKA), cAMP responsive elements (CREB), and PGC1α pathway, which is overlapped with adaptive thermogenesis triggered by β-adrenalin receptor agonists^17–20^.

We have reported chemical compound-induced brown adipocytes, ciBAs, converted from human dermal fibroblasts using a combination of small molecules without using transgene^21^. We further optimized the chemical combination and developed a serum-free brown adipogenic medium (SFBAM) for the direct conversion^22^. ciBAs exhibited several distinct features of brown/beige adipocytes such as increased mitochondrial levels, elevated oxygen consumption rates, and a response to β-adrenergic receptor agonists, and increased expression of a specific set of adipocyte-enriched genes^22^. However, the expression of a broad range of other metabolic and fibrogenic genes remained largely unknown. To reveal the molecular mechanism underlying the direct conversion into ciBAs from human dermal fibroblasts, transcriptome analysis was performed by RNA-Seq approach. Moreover, transcriptional changes in the direct conversion were compared with those of the adipocyte browning stimulated by Forskolin in AdMSC to characterize the underlying mechanism.

## Results

### Transcriptome analysis in the direct conversion into ciBAs

**Figure 1:**
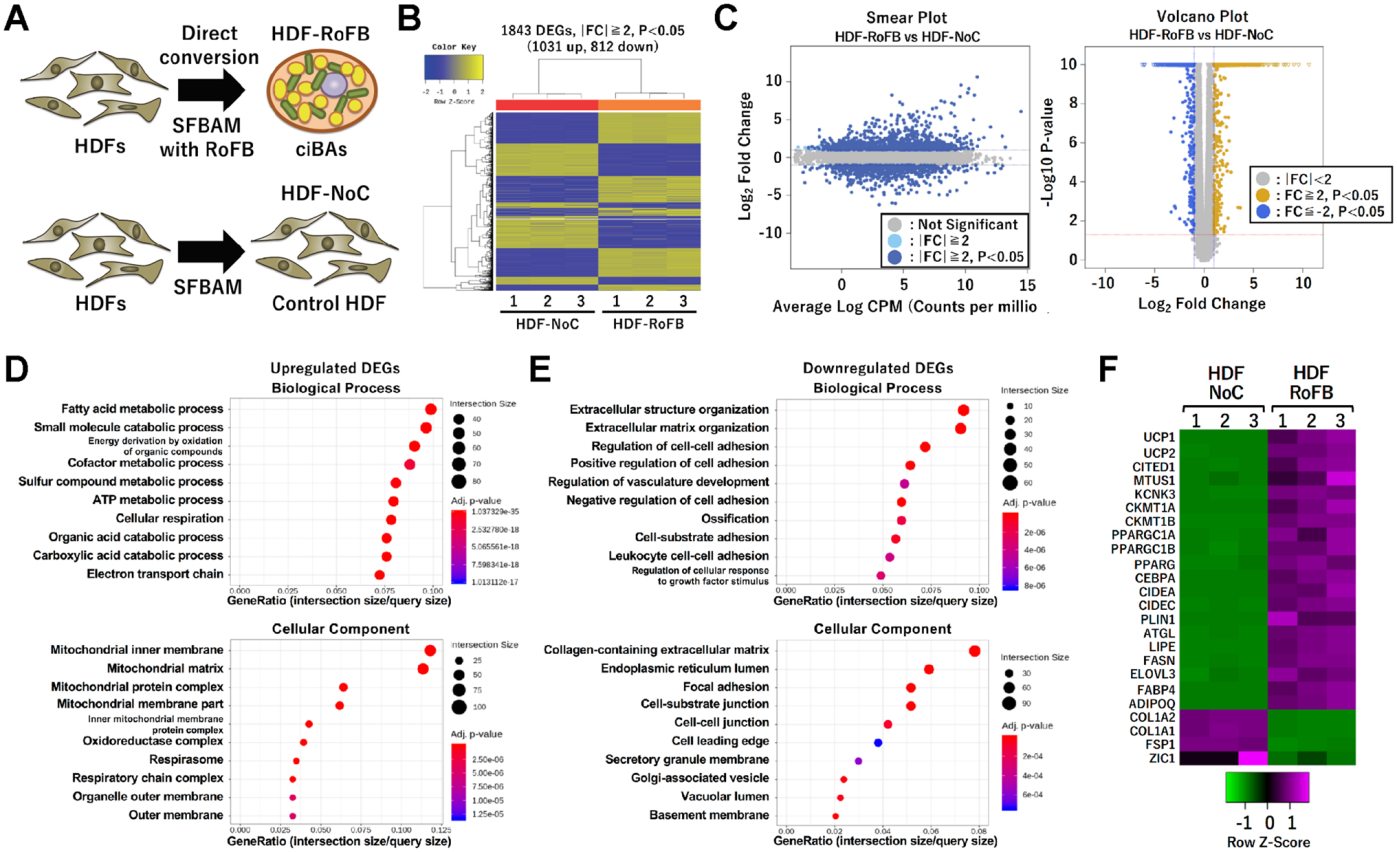
Genome-wide transcriptional analysis of ciBAs. (**A**) A schematic figure indicates human dermal fibroblasts were treated with either RoFB (HDF-RoFB) or no compound (HDF-NoC) in the serum-free brown adipogenic medium, SFBAM. (**B**) Heat map and hierarchical clustering analysis represent 1843 differentially expressed genes (DEGs) (|FC| ≧ 2, P < 0.05) between HDF-NoC and HDF-RoFB. (**C**) Smear and Volcano plots indicate logarithmic fold change, P-value, and CPM (counts per million) obtained from the comparison between HDF-NoC and HDF-RoFB. (**D**,**E**) Gene ontology (GO) enrichment analysis was performed in the up-regulated DEGs (**D**) and the down-regulated DEGs (**E**). Top 10 GO terms are represented in the categories of Biological Process and Cellular Component. (**F**) A heat map analysis shows z-scored FPKM obtained the RNA-Seq results in adipocytes-enriched and fibrogenic genes that we have previously analyzed by qRT-PCR.

To reveal comprehensive transcriptional changes in the chemical conversion, RNA-Seq analysis was performed in both ciBAs (HDF-RoFB) and the control fibroblasts (HDF-NoC) (Fig. 1A). ciBAs were induced for 3 weeks by culturing human dermal fibroblasts (HDF38) with the chemical cocktail consisting of RoFB in the SFBAM. The control fibroblasts were cultured in the SFBAM without RoFB in parallel. In the result, 44–65 million reads were acquired in these samples, of which approximately 98% were mapped into the human reference genome (Table 1). The quality of base calling as represented by the Phred score Q30 was sufficiently high (about 95%). The heat map indicated that in total 1843 differentially expressed genes (DEGs) between HDF-RoFB and HDF-NoC were identified (Fig. 1B). The hierarchical clustering in the heat map analysis graphically represents the similarity of gene expression patterns between samples and genes. The smear and volcano plots represented that these DEGs with over twofold changes were distributed with widespread read counts and P-values (Fig. 1C). Gene ontology (GO) enrichment analysis suggested that the upregulated DEGs were most significantly categorized into functional groups related to energy metabolism and mitochondria (Fig. 1D). In contrast, the down-regulated DEGs were enriched in fibroblast-related functions such as extracellular matrix and cell adhesion (Fig. 1E). To verify whether the RNA-Seq results were valid, we checked major adipocyte-enriched genes previously analyzed by qRT-PCR^22^ (Fig. 1F). The heat map indicated that the RNA-Seq results well reflected the expression in our previous report. The GO enrichment analysis indicated that the DEGs were closely associated with characteristic features in brown/beige adipocytes.

**Table 1:** Summary of RNA-Seq analysis in ciBAs and AdMSC-derived adipocytes. Total number of bases sequenced, total number of reads, overall read mapping ratio (%), GC content (%), Q20* (%), and Q30** (%) were calculated.

Sample ID	Total read bases	Total reads	Mapped reads (%)	GC (%)	Q20 (%)	Q30 (%)
HDF-NoC-1	5,600,507,461	55,689,494	97.91	51.68	98.89	95.96
HDF-NoC-2	5,298,964,192	52,704,944	98.38	52.30	98.92	96.07
HDF-NoC-3	5,973,476,718	59,380,808	98.57	51.98	98.93	96.07
HDF-RoFB-1	5,720,846,941	56,893,694	98.49	51.63	98.85	95.88
HDF-RoFB-2	6,568,741,794	65,379,548	98.44	51.62	98.49	94.84
HDF-RoFB-3	4,466,180,430	44,413,992	98.67	51.44	98.87	95.92
AdMSC-NoC-1	4,914,895,330	48,662,330	97.51	51.77	98.81	96.18
AdMSC-NoC-2	5,071,207,374	50,209,974	96.99	51.79	98.81	96.19
AdMSC-NoC-3	4,594,737,450	45,492,450	97.42	52.09	98.65	95.80
AdMSC-Ro-1	4,797,708,693	47,704,436	98.38	52.13	98.93	96.08
AdMSC-Ro-2	4,245,978,419	42,227,554	98.01	52.46	98.87	95.95
AdMSC-Ro-3	4,374,445,978	43,504,956	98.41	52.53	98.91	96.07
AdMSC-RoFB-1	4,459,469,029	44,357,190	98.26	52.15	98.87	95.96
AdMSC-RoFB-2	5,128,410,496	50,983,450	98.30	51.27	98.95	96.11
AdMSC-RoFB-3	4,384,356,023	43,591,650	98.23	51.85	98.98	96.22

*% of bases with quality over Phred score 20.

**% of bases with quality over Phred score 30.

### ciBAs reprogram energy and mitochondrial metabolism

**Figure 2:**
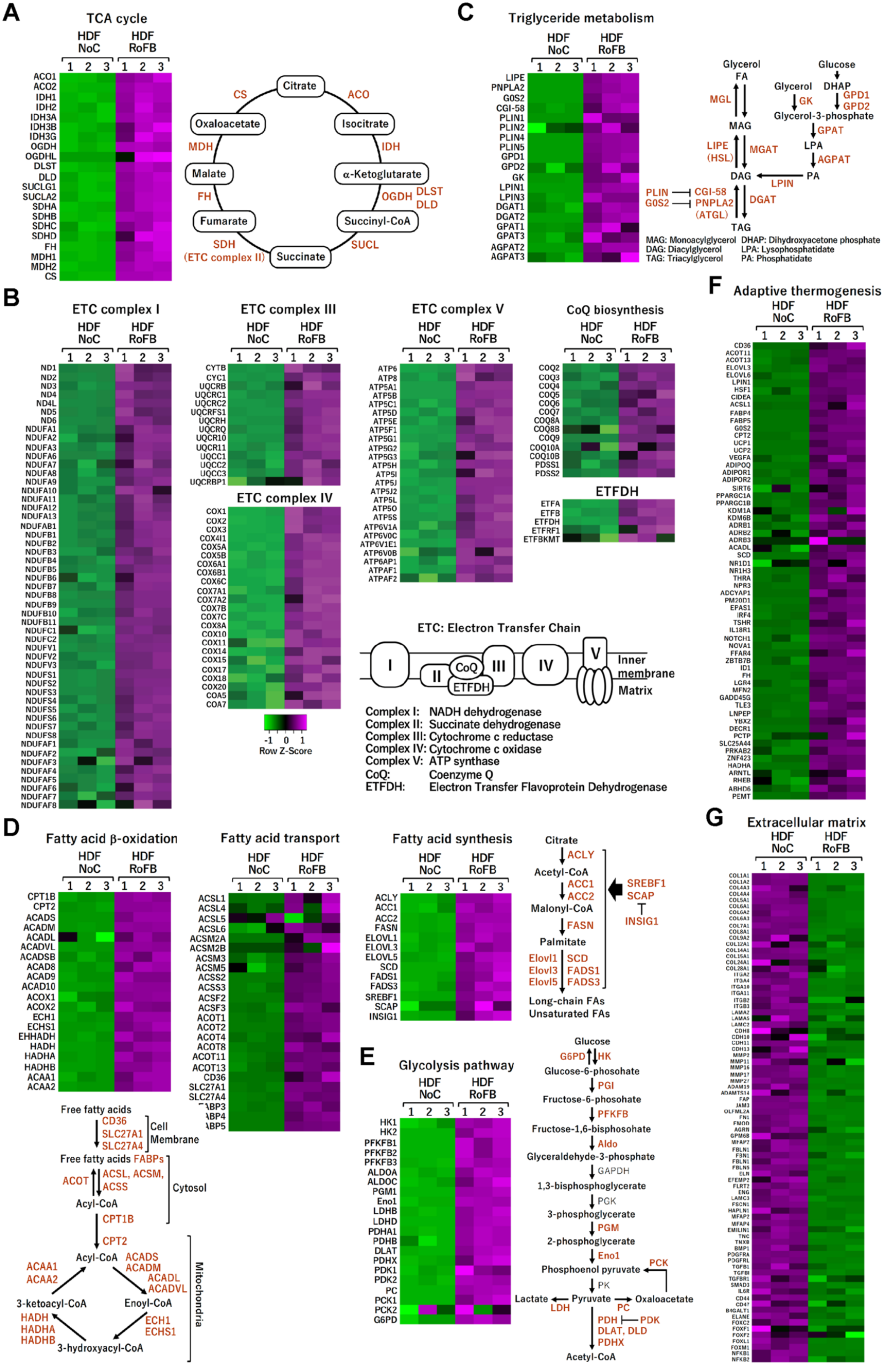
Heat map analysis of transcriptional profiles in ciBAs. (**A**–**G**) The transcriptional profiles between HDF-NoC and HDF-RoFB are shown as heat maps in functional groups such as TCA cycle (**A**), mitochondrial ETC complex (**B**), triglyceride metabolism (**C**), fatty acid β-oxidation, transport, and synthesis (**D**), glycolysis pathway (**E**), adaptive thermogenesis (**F**), and extracellular matrix (**G**). The color scale shows z-scored FPKM representing mRNA levels of each gene in green (lower expression) and magenta (higher expression). Schematic figures show each metabolic pathway including the up-regulated genes (highlighted by reddish brown) listed in the heat maps.

We further pursued transcriptional changes of ciBAs in each metabolic pathway. The heat map analysis exhibited enhanced expression of a series of enzymatic genes in tricarboxylic acid (TCA) cycle in ciBAs (HDF-RoFB) compared to the control fibroblasts (HDF-NoC) (Fig. 2A). In addition, most genes of mitochondrial electron transfer chain (ETC) complex I–V, encoded in both genomic and mitochondrial DNA, were consistently up-regulated (Fig. 2B). The expression of a series of other mitochondria-related genes was also increased in mitochondrial ribosomes, antioxidant enzymes, and apoptosis (Supplementary Fig. S1). Consistent with the results of GO analysis, ciBAs highly expressed numerous metabolic genes involved in both triglyceride lipolysis and synthesis (Fig. 2C), fatty acid β-oxidation, transport, and synthesis (Fig. 2D). Glycolysis pathway was also activated in ciBAs (Fig. 2E). In addition, the genome-wide transcriptional analysis identified more genes related to adaptive thermogenesis (Fig. 2F). In contrast, ciBAs suppressed a broad range of fibrogenic genes such as collagens, integrins, laminins, cadherins, and fibronectins (Fig. 2G). These RNA-Seq results suggested that the cell fate change from dermal fibroblasts to brown adipocyte-like cells was advanced in a genome-wide manner.

### Characterization of adipocyte browning stimulated by Forskolin in AdMSC

**Figure 3:**
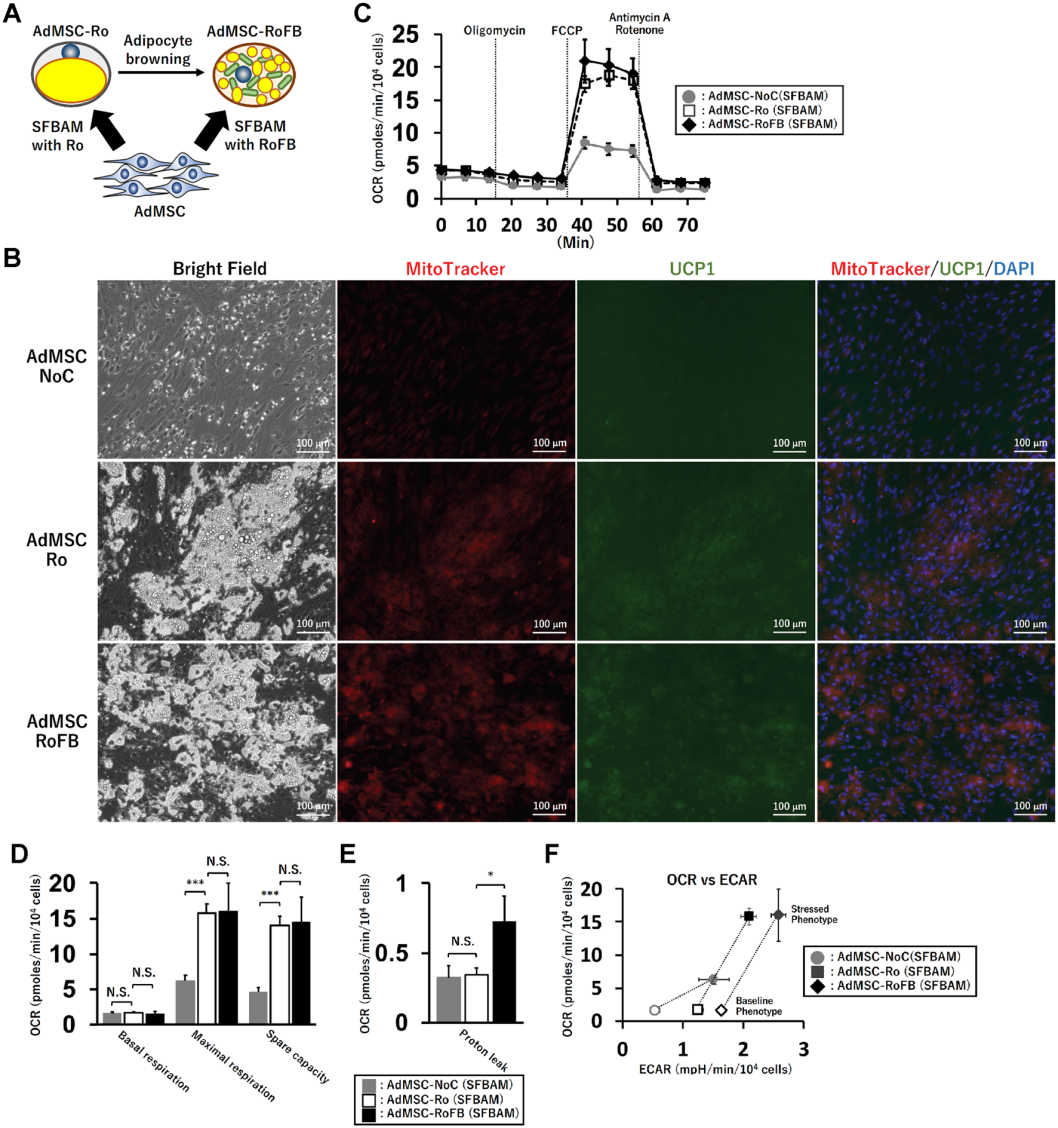
Characterization of AdMSC-derived adipocytes. (**A**) A schematic figure indicates that two types of adipocytes were differentiated from AdMSC by the treatment with either Ro (AdMSC-Ro) or RoFB (AdMSC-RoFB) in SFBAM. (**B**) Representative images of bright field, mitochondrial labelling with MitoTracker (red), UCP1 protein expression (green), and merged image in the control AdMSC (AdMSC-NoC) and the AdMSC-derived adipocytes (AdMSC-Ro and AdMSC-RoFB). The nuclei were visualized by DAPI (blue). Scale bars represent 100 μm. (**C**) Oxygen consumption rate (OCR) was measured using the Flux analyzer in AdMSC-NoC (grey circles), AdMSC-Ro (open squares), and AdMSC-RoFB (black diamonds). Oligomycin, FCCP, and Antimycin A/Rotenone were added during the measurement as indicated. (**D**,**E**) Each OCR corresponding to basal respiration, maximal respiration, and spare capacity (**D**), and proton leak (**E**) was calculated. (**F**) Energy phenotype profile in AdMSC-NoC (grey circles), AdMSC-Ro (open squares), and AdMSC-RoFB (black diamonds). OCR and extracellular acidification rate (ECAR) were plotted under basal (open circle, square, and diamond) and stressed (closed circle, square, and diamond) conditions. The stressed condition was caused by the treatment with both Oligomycin and FCCP. Data represent mean ± SEM (n = 6). Student’s t-test: *P < 0.05, ***P < 0.001, *N.S.* not significant.

Next, we used AdMSC as a control for adipocyte browning (Fig. 3A). AdMSC derived from a human subject at age 46 years (AdMSC46) was differentiated into two types of adipocytes by treatment with either Rosiglitazone only (AdMSC-Ro) or the RoFB cocktail (AdMSC-RoFB), the same chemical combination as ciBAs. Bright field images indicated that the condition of AdMSC-Ro and AdMSC-RoFB was efficiently differentiated into adipocytes with lipid droplets compared to the control AdMSCs cultured in parallel with SFBAM only (AdMSC-NoC) (leftmost panels of Fig. 3B). The differentiation efficiency of AdMSC was estimated at about 60%, while the conversion efficiency of ciBAs from HDF was about 40% (Supplementary Fig. S2A). Immunocytochemical analysis indicated that the AdMSC-derived adipocytes enhanced cellular mitochondrial signals (left middle panels of Fig. 3B). Consistently the mitochondrial content was also increased in these adipocytes (Supplementary Fig. S2B). UCP1 protein was only slightly stained in both AdMSC-derived adipocytes (right middle panels of Fig. 3B). However, UCP1 was more clearly stained in ciBAs (Supplementary Fig. S2C), implicating that the expression level might be potentially low.

To further characterize the two types of the AdMSC-derived adipocytes, oxygen consumption rate (OCR) was measured by Seahorse XFe96 extracellular flux analyzer. The OCR was typically varied by adding the inhibitors for mitochondrial ETC (Fig. 3C). The OCR corresponding to the maximal respiration and the spare capacity was increased in both AdMSC-Ro and AdMSC-RoFB adipocytes compared to AdMSC-NoC, but not much different between these adipocytes (Fig. 3D). The OCR corresponding to the proton leak was significantly increased in AdMSC-RoFB (Fig. 3E and Supplementary Table S1). The energy phenotype profile showed that the AdMSC-derived adipocytes exhibited a higher metabolic potential particularly under the stressed condition (Oligomycin + FCCP) compared to the control AdMSCs (Fig. 3F). Extracellular acidification rates (ECAR) were a little increased between AdMSC-RoFB and AdMSC-Ro under both the basal and stressed conditions.

### Transcriptome analysis of the adipocyte browning in AdMSC

**Figure 4:**
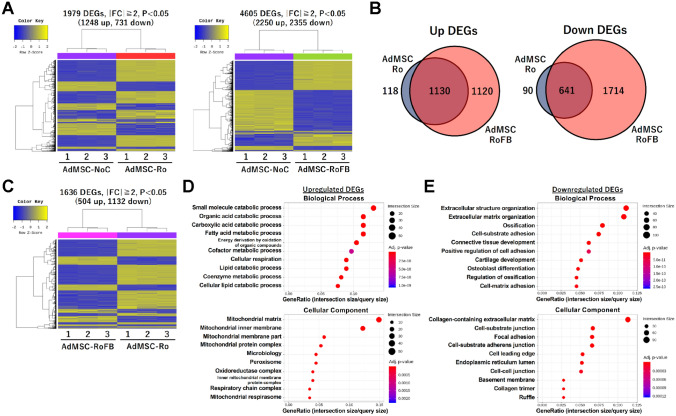
Genome-wide transcriptional analysis of AdMSC-derived adipocytes. (**A**) Heat map and hierarchical clustering analyses represent 1979 DEGs (|FC| ≧ 2, P < 0.05) and 4605 DEGs in AdMSC-Ro and AdMSC-RoFB, respectively, compared to AdMSC-NoC. The DEGs in AdMSC-Ro consist of 1248 up- and 731 down-regulated genes, while the DEGs in AdMSC-RoFB consist of 2250 up- and 2355 down-regulated genes. (**B**) Venn diagrams represent the overlap of the up- and down-regulated DEGs in AdMSC-Ro and AdMSC-RoFB. (**C**) Heat map and hierarchical clustering analysis represent 1636 DEGs (|FC| ≧ 2, P < 0.05) between AdMSC-RoFB and AdMSC-Ro. The DEGs consist of 504 up- and 1132 down-regulated genes. (**D**,**E**) GO analysis was performed by means of DAVID in the up-regulated DEGs (**D**) and the down-regulated DEGs (**E**). Top 10 GO terms were shown in the categories of Biological Process and Cellular Component.

RNA-Seq analysis was also performed in the AdMSC-derived adipocytes (Table 1). Compared with the results of the control AdMSC (AdMSC-NoC), 1248 up- and 731 down-regulated genes were identified in AdMSC-Ro, whereas 2250 up- and 2355 down-regulated genes were identified in AdMSC-RoFB (Fig. 4A). Venn diagrams represented that most of the up- and down-regulated genes in AdMSC-Ro were overlapped with those in AdMSC-RoFB (Fig. 4B), implicating that the treatment with Forskolin collaboratively activated gene expression without disturbing the effects of Rosiglitazone. Next, to identify transcriptional changes in the adipocyte browning of AdMSC, the RNA-Seq results were compared between AdMSC-RoFB and AdMSC-Ro adipocytes. The heat map showed that 504 up- and 1132 down-regulated genes were identified (Fig. 4C). The smear and volcano plots showed that the DEGs with over twofold changes were properly distributed with widespread CPM (counts per million) and P-values (Supplementary Fig. S3). GO enrichment analysis indicated that the up-regulated DEGs were most significantly grouped into fatty acid metabolism and mitochondria (Fig. 4D), while the down-regulated DEGs were enriched in extracellular structure and matrix (Fig. 4E), suggesting that these GO terms highly resembled ciBAs.

**Figure 5:**
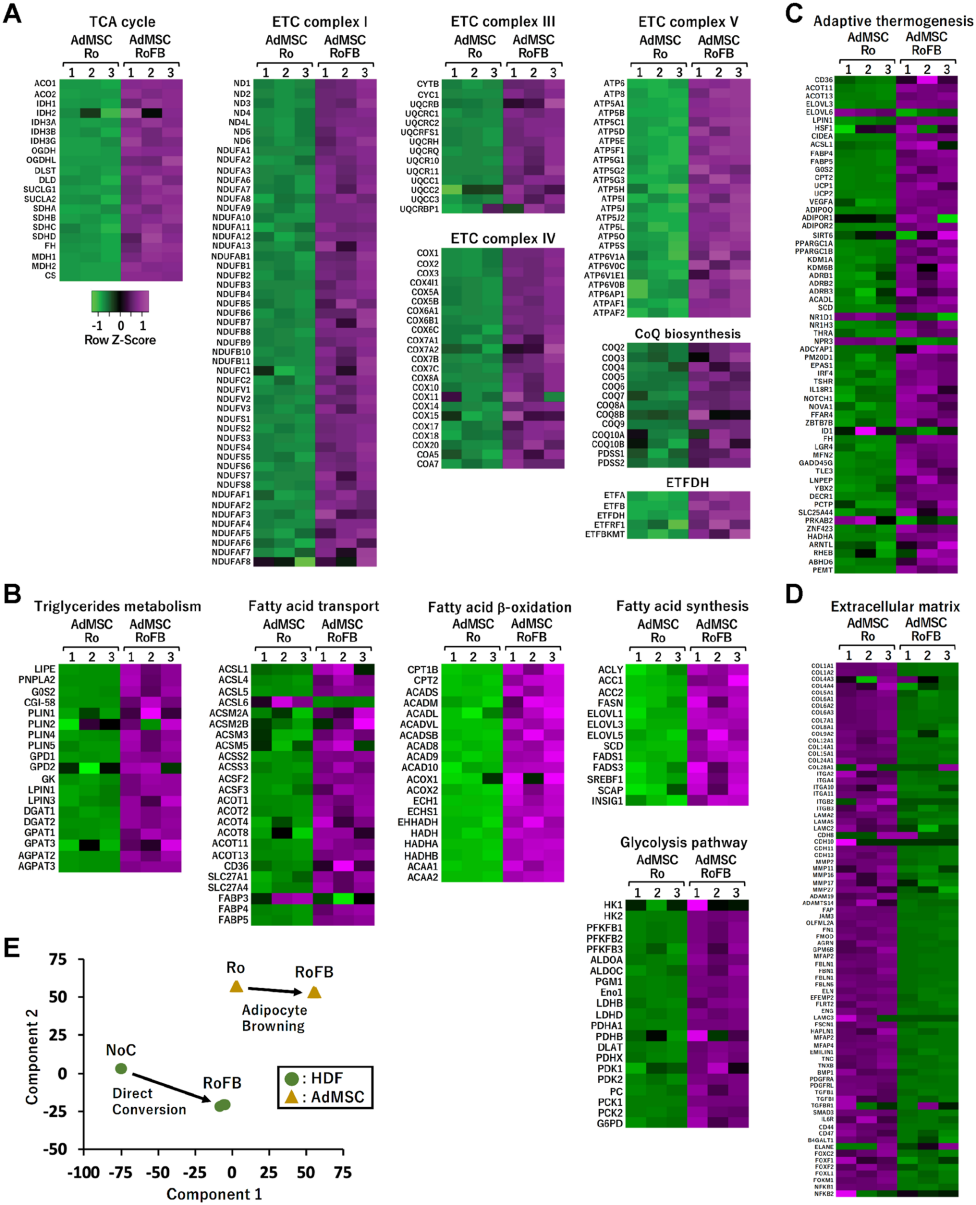
Heat map analysis of transcriptional profiles in AdMSC-derived adipocytes. (**A–D**) The transcriptional profiles between AdMSC-Ro and AdMSC-RoFB are shown as heat maps in the same set of genes as shown in Fig. 2. The color scale shows z-scored FPKM representing mRNA levels of each gene in green (lower expression) and magenta (higher expression). (**E**) Multidimensional scaling analysis graphically indicates the similarity and variability of total expression patterns of HDF- and AdMSC-derived samples as indicated.

The expression in the same set of metabolic and mitochondria-related genes as ciBAs was compared by heat map analysis between AdMSC-Ro and AdMSC-RoFB. The results represented that most of the genes involved in the TCA cycle, ETC complexes (Fig. 5A), triglycerides and fatty acid metabolism (Fig. 5B), and adaptive thermogenesis (Fig. 5C) were enhanced in AdMSC-RoFB. Almost the same set of genes involved in extracellular matrix were repressed (Fig. 5D). Multidimensional scaling analysis visually suggested that the component 1 axis mainly reflected transcriptional changes between AdMSC-Ro and AdMSC-RoFB, corresponding to the adipocyte browning, while the component 2 axis might reflect dissimilarities between HDFs and AdMSCs. It is likely that the change of the component 1 axis between HDF-NoC and HDF-RoFB indicated similarities between the direct conversion into ciBAs and the adipocyte browning of AdMSCs.

### Differential expression pattern of *Ucp1* and *Ucp2* between ciBAs and the AdMSC-derived adipocytes

**Figure 6:**
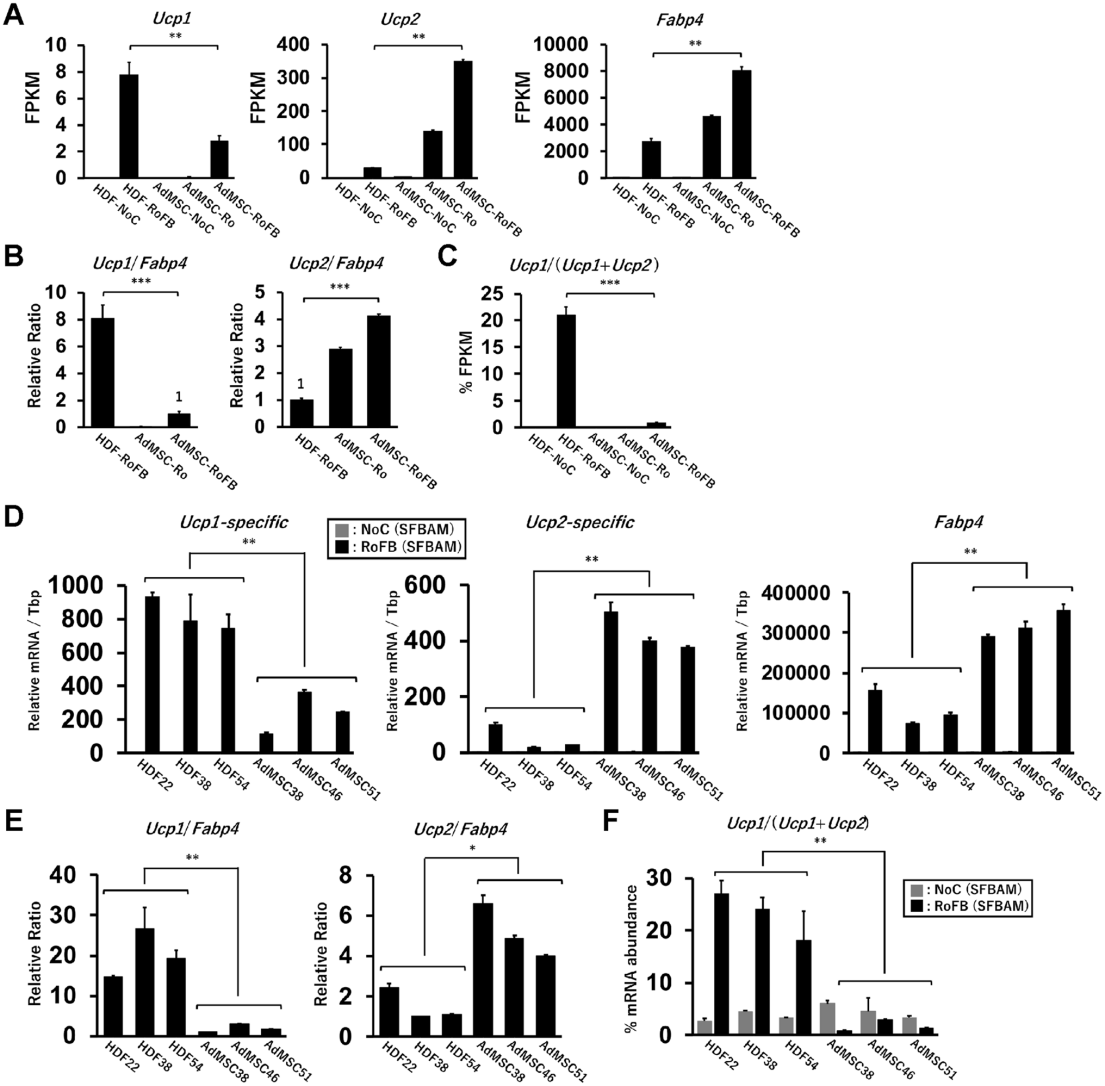
Transcriptional analysis of *Ucp1* and *Ucp2* in ciBAs and AdMSC-derived adipocytes. (**A**) FPKM values in *Ucp1*, *Ucp2*, and *Fabp4* were obtained from the RNA-Seq data. (**B**) The ratios of *Ucp1* and *Ucp2* FPKM to *Fabp4* FPKM are represented. (**C**) Percent FPKM values of *Ucp1* in *Ucp1* and *Ucp2* mRNAs were calculated. (**D**) The expression of *Ucp1*, *Ucp2*, and *Fabp4* was quantified by qRT-PCR in ciBAs converted from three different lines of human dermal fibroblasts and adipocytes differentiated from three different lines of AdMSCs under the same culture condition. The PCR primers for *Ucp1* and *Ucp2* were designed to selectively amplify each mRNA. Grey bars indicate the control with SFBAM only, while black bars indicate the results of either ciBAs or the AdMSC-derived adipocytes induced with RoFB in SFBAM. (**E**) The ratios of *Ucp1* and *Ucp2* mRNA to *Fabp4* mRNA are represented. (**F**) Percent mRNA abundance of *Ucp1* in *Ucp1* and *Ucp2* mRNAs was calculated by using DNA templates whose copy numbers were calculated for the quantification. Data represent mean ± SD (n = 3). Student’s t-test: *P < 0.05, **P < 0.01, ***P < 0.001.

The expression levels of major adipogenic genes including *Ppargc1b*, *Pparg*, *Cebpa*, *Plin1*, *Atgl*, *Lipe*, *Pnpla2*, *Fasn*, *Cidec*, *Cidea*, *Elovl3*, and *AdipoQ* in the AdMSC-derived adipocytes were higher than ciBAs (Supplementary Fig. S4). The RNA-Seq results also revealed that *Ucp1* mRNA was more expressed in ciBAs (HDF-RoFB) while *Ucp2* mRNA was more abundant in the AdMSC-derived adipocytes (AdMSC-Ro and AdMSC-RoFB) (Fig. 6A). The amino acid sequence of UCP1 protein is highly homologous to that of UCP2 (75% similarity, 59% identity). The RNA-Seq analysis distinguished transcripts derived from each *Ucp1* and *Ucp2* gene. A higher differentiation efficiency in AdMSC compared to ciBAs was estimated based on the expression level of *Fabp4*, one of the adipocyte-enriched genes. According to our previous studies, the ratio of *Ucp1* to *Fabp4* was calculated to evaluate adipocyte browning in these adipocytes. Under the same experimental condition, the ratio in ciBAs (HDF-RoFB) was about 8 times higher than that in AdMSC-RoFB adipocytes (Fig. 6B). Consistent with our hypothesis, the ratio was very low in AdMSC-Ro adipocytes. In contrast, the ratio of *Ucp2* to *Fabp4* in AdMSC-RoFB adipocytes was about 4 times higher than that in ciBAs. The percent ratio of *Ucp1* mRNA level in *Ucp1* and *Ucp2* mRNAs was about 20% in ciBAs, whereas it was a few percent in AdMSC-RoFB adipocytes (Fig. 6C).

To precisely measure the differential expressional pattern of *Ucp1* and *Ucp2* mRNA, real time QPCR analysis was performed in three different lines of HDFs and AdMSCs (Supplementary Table S2). To distinguish *Ucp1* mRNA from *Ucp2* mRNA, the PCR primers were carefully designed to the specific regions of each *Ucp* mRNA (Supplementary Fig. S5). In the results, *Ucp1-specific* mRNA levels in ciBAs converted from these HDFs were significantly higher than those of the adipocytes differentiated from these AdMSCs (Fig. 6D). In contrast, *Ucp2-specific* mRNA levels were higher in the AdMSC-derived adipocytes. Each *Ucp* mRNA normalized by *Fabp4* mRNA also indicated higher expression pattern of *Ucp1* in ciBAs than the AdMSC-derived adipocytes (Fig. 6E). The *Ucp1* percent ratio in *Ucp1* and *Ucp2* mRNAs was about 20% in ciBAs while it was only a few percent in the AdMSC-derived adipocytes (Fig. 6F). These results were well consistent with the RNA-Seq results.

**Figure 7:**
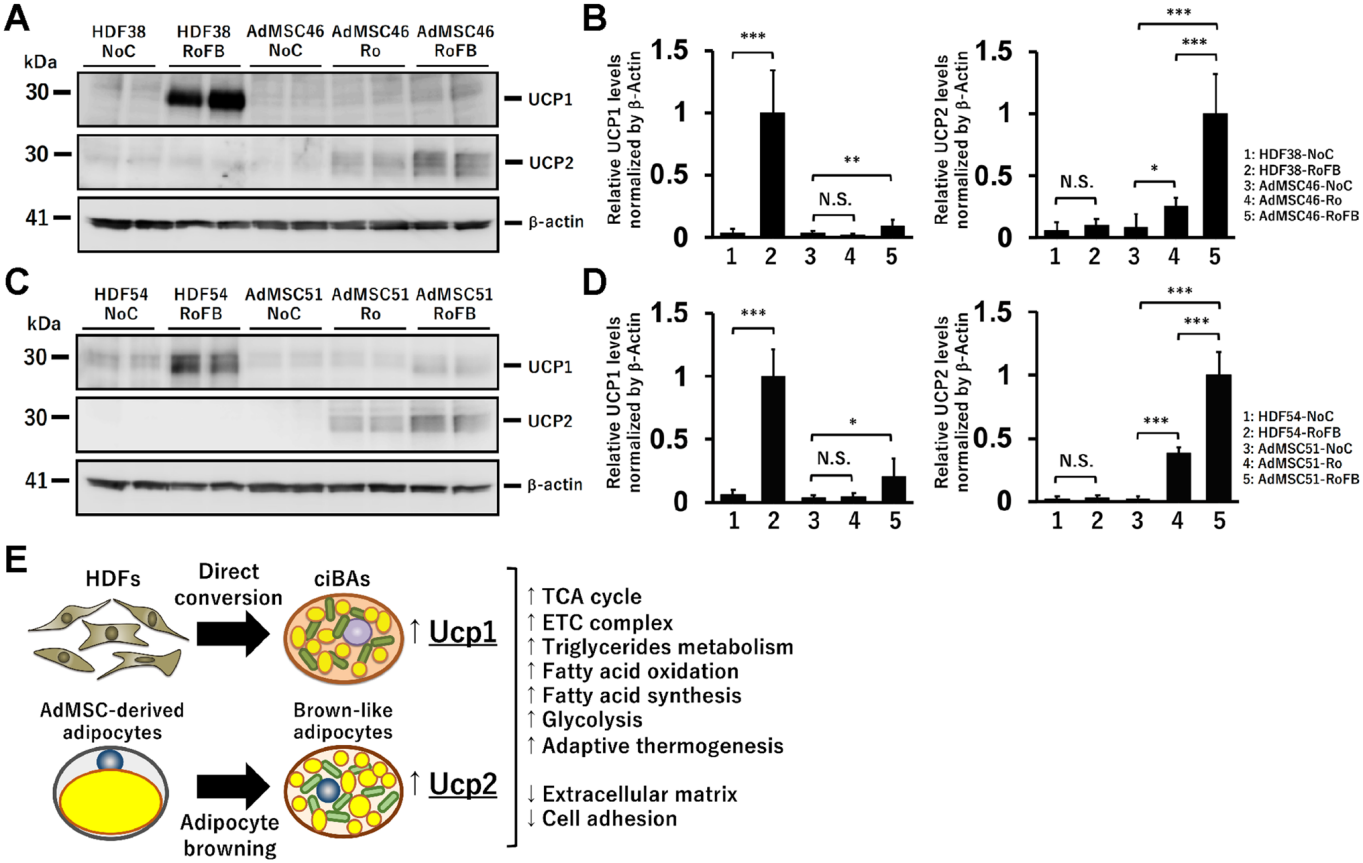
Comparative expression pattern of UCP1 and UCP2 proteins in ciBAs and AdMSC-derived adipocytes. (**A**) The protein levels of UCP1 and UCP2 were quantified by immunoblotting in the adipocytes derived from HDF38 and AdMSC46. (**B**) The band intensities were measured by densitometry using ImageJ software. Full length blots are presented in Supplementary Fig. S7. β-Actin protein was used as a loading control for normalization. Data represent mean ± SD (n = 6). Student’s t-test: *P < 0.05, **P < 0.01, ***P < 0.001, *N.S.* not significant. (**C**,**D**) The protein levels of UCP1 and UCP2 were quantified in a different set of the adipocytes derived from HDF54 and AdMSC51. (**E**) A summary of transcriptome analysis in the comparison between the direct conversion to ciBAs and the adipocyte browning of AdMSC. In both biological pathways, a series of genes were activated in functional groups such as related to energy metabolism, mitochondria, and adaptive thermogenesis, while many fibrogenic genes involved in extracellular matrix and cell adhesion were repressed. These results suggested that the direct conversion to ciBAs underwent genome-wide transcriptional changes similar to the adipocyte browning of AdMSC. One of the most characteristic differences between them was the expression pattern of major uncoupling proteins. ciBAs more strongly expressed *Ucp1* responsible for adrenergic thermogenesis in brown/beige adipocytes, while the AdMSC-derived adipocytes predominantly expressed *Ucp2* with a weaker uncoupling activity and other functions.

Western blotting analysis represented that UCP1 protein was expressed in ciBAs (HDF-RoFB) at a higher level than the AdMSC-derived adipocytes (AdMSC-Ro and AdMSC-RoFB) (Fig. 7A,B). UCP2 protein was mainly detected in the AdMSC-derived adipocytes, indicating that the protein expression was reflected by the difference of *Ucp1* and *Ucp2* mRNA levels. A different set of HDF and AdMSC also exhibited similar results of the expression pattern of UCP1 and UCP2 proteins (Fig. 7C,D). The protein stability of UCP1 in ciBAs might be a little higher than AdMSC-RoFB adipocytes, but they were not so dramatically different (Supplementary Fig. S6). These results suggested that the expression of major mitochondrial uncoupling proteins was different between ciBAs and the AdMSC-derived adipocytes under the same experimental condition (Fig. 7E).

## Discussion

In this study, we successfully identified transcriptional profiles for brown adipogenic reprogramming in the direct conversion from human dermal fibroblasts into ciBAs. ciBAs induced by a chemical cocktail, RoFB, in the serum-free medium showed enhanced expression of multiple genes closely associated with the functions in brown/beige adipocytes (Fig. 7E). The activation of the fatty acid and glucose metabolic genes was likely associated with elevated oxygen consumption and glycolysis rates in ciBAs as reported previously^22^. Moreover, many fibrogenic genes involved in extracellular matrix were transcriptionally repressed, implying that the cell fate of dermal fibroblasts was converted in a genome-wide manner. A part of the gene regulations has already been detected in our previous reports^21,22^. In this study, the transcriptome analysis comprehensively demonstrated that ciBAs underwent integrated changes of gene expression in multiple functional groups (Fig. 2). Another purpose of this study is to compare the direct conversion with the adipocyte browning stimulated by the treatment with Forskolin in the primary culture of adipose tissue-derived MSC. The transcriptomic analysis revealed that almost the same sets of the genes as the direct conversion were either activated or repressed in the process of the adipocyte browning (Fig. 5). These results provided more insights into the molecular mechanism underlying the direct conversion, which is fundamental to the application of ciBAs for basic research, drug development, and other clinical uses.

This study also proposed that one of the most characteristic differences between ciBAs and the AdMSC-derived adipocytes was the expression pattern of mitochondrial uncoupling proteins. Both RNA-Seq and qRT-PCR results showed that *Ucp1* mRNA levels were higher in ciBAs while *Ucp2* mRNA was more abundant in the AdMSC-derived adipocytes (Fig. 6). A similar tendency was observed in three different lines of HDFs and AdMSCs. UCP1 protein was expressed at the same or higher levels than the difference of the mRNA in ciBAs (Fig. 7), which might be due to post-transcriptional and post-translational regulations^23,24^. A little higher stability of UCP1 protein might partially explain the higher expression in ciBAs (Supplementary Fig. S6). This was further supported by the observation that immunostaining of UCP1 protein was only slightly detected in the AdMSC-derived adipocytes (Fig. 3B). A number of studies on gene targeting of *Ucp1* have suggested that UCP2 is not able to compensate for thermogenic functions in brown adipocytes^25,26^.

*Ucp1* is highly abundant in brown and beige adipocytes in vivo, while *Ucp2* is ubiquitously expressed across various tissues and cell types^27,28^. Gene targeting and RNA interference approaches have indicated that UCP2 has a weak uncoupling activity, but UCP2 is physiologically required for different functions such as regulation of mitochondrial oxidative stress and energy metabolism^29,30^. In this study, both AdMSC-derived adipocytes (AdMSC-Ro and AdMSC-RoFB) showed increased OCR corresponding to the maximal respiration, but not the basal respiration (Fig. 3D). In contrast, the proton leak activity was increased in the AdMSC-RoFB adipocytes only (Fig. 3E), while the proton leak activity was not changed in AdMSC-Ro adipocytes in spite of the increased *Ucp2* expression.

AdMSCs are also referred to as preadipocytes including non-thermogenic adipocyte progenitor subtypes^16^. The AdMSC-derived adipocytes expressed adipocyte-enriched genes to a greater extent than ciBAs (Supplementary Fig. S4). Although the higher expression levels were partially due to a higher differentiation efficiency in AdMSC (Supplementary Fig. S2A), the adipocytes might possess more features of white adipocytes rather than brown ones. In addition, the mitochondrial content of AdMSC-RoFB adipocytes was a little higher than we reported previously in ciBAs^22^ (Supplementary Fig S2B). But the differentiation efficiency in AdMSC-RoFB was also higher by about 1.5 times, so the content should be almost comparable between them. It should be noted that different components in the adipogenic medium and differentiation protocols might provide more brown phenotypes including higher *Ucp1* expression in AdMSC-derived adipocytes. Further studies using isogenic and more HDF and AdMSC lines are also required to precisely conclude which cell types are superior for a model of human beige adipocytes. In this study, the continuous stimulation by Forskolin further induced the expression of energy metabolic and mitochondria-related genes including *Ucp1* (Fig. 5), indicating that Forskolin promotes adipocyte browning. The induction of cellular cAMP by Forskolin activates PKA-dependent phosphorylation of CREB and P38 MAP kinase-mediated phosphorylation of ATF2^17,20^. The phosphorylated CREB and ATF2 directly and indirectly activated *Ucp1* transcription. Such a pathway via cAMP is partially overlapped by adrenergic thermogenesis pathway during cold exposure^18,20^. Thus, our study also provided more insights into transcriptional effects of Forskolin on the adipocyte browning, which mimics adrenergic stimulation during cold exposure.

It is still difficult to isolate homogenous primary human beige (pre)adipocytes sporadically dispersed in white adipose depots. A proper model for human beige adipocytes has been required to identify small molecules and cytokines promoting brown adipogenesis^31^. Such bioactive molecules might have a therapeutic potential for the prevention of obesity and related metabolic diseases by controlling systemic energy balance. Taken together, our experimental results revealed transcriptional similarities and differences between ciBAs and the AdMSC-derived adipocytes. We propose that ciBAs will become a promising model for human beige adipocytes for basic research, drug development, and future clinical applications.

## Methods

### Cell culture

Human dermal fibroblasts were purchased from DS Pharma Biomedical Co. (Osaka, Japan) as reported previously^22^. AdMSCs were purchased from Takara (C-12977, Takara, Shiga, Japan). The information is listed in Supplementary Table S2. The fibroblasts derived from a human subject at age 38 years (HDF38) were mainly used for RNA-Seq analysis and other experiments. About 1.5 × 10^5^ cells were seeded on a 35-mm dish with high-glucose DMEM (11995-065, ThermoFisher Scientific, MA, USA) supplemented with 10% FBS (HyClone, UT, USA) and penicillin/streptomycin (ThermoFisher Scientific). After reaching 80–90% confluence, the medium was changed to start direct conversion into ciBAs with the SFBAM prepared from high-glucose DMEM (11995-065) supplemented with 3,3′,5 Triiodothyronine (T3) (Sigma-Aldrich, MO, USA), Dexamethasone (FUJIFILM Wako, Osaka, Japan), 3-isobutyl-1-methylxanthine (IBMX) (FUJIFILM Wako), human recombinant insulin (FUJIFILM Wako), l-ascorbic acid-2-phosphate (Sigma-Aldrich), linoleic acid- and oleic acid-albumin (L9655-5ML, Sigma-Aldrich) and penicillin/streptomycin (ThermoFisher Scientific). The final concentrations of these components have been previously reported^22^. The combination of RoFB consists of Rosiglitazone (1 μM), Forskolin (7.5 μM), and human recombinant BMP7 (20 ng/ml). For the direct conversion, human fibroblasts were incubated with SFBAM including the chemical cocktail for 3 weeks unless otherwise indicated. The AdMSCs derived from a human subject at age 46 years (AdMSC46) were mainly used for RNA-Seq analysis and other experiments. AdMSC was cultured in Mesenchymal Stem Cell Growth Medium 2 (Takara). After reaching 80–90% confluence, AdMSCs were differentiated into adipocytes with SFBAM including either Rosiglitazone or RoFB for 2 weeks. All experimental procedures were conducted in accordance with the general regulations in Kyoto Prefectural University of Medicine.

### RNA-sequencing

Total RNA was prepared from the control fibroblasts (HDF-NoC) and ciBAs (HDF-RoFB) derived from HDF38 by FastGene RNA premium kit (Nippon Genetics, Tokyo, Japan). Total RNA was also prepared from the control AdMSCs (AdMSC-NoC) and the adipocytes differentiated from AdMSC46 by the treatment with either Ro (AdMSC-Ro) or RoFB (AdMSC-RoFB). RNA integrity number (RIN) values were > 9 in all the RNA samples. The library was prepared by TruSeq stranded mRNA LT Sample Prep Kit (Illumina, CA, USA), following the manufacturer’s low sample (LS) protocol. 100 bp paired-end sequencing was performed by NovaSeq 6000 System (Illumina). FASTQ files were created for each sample using bcl2fastq conversion software (Illumina). Trimmomatic program was used to remove adapter sequences and bases with base quality lower than three from the ends. Trimmed reads were mapped to a reference genome (NCBI GRCh37) with HISAT2. After the read mapping, StringTie was used for transcript assembly. After the assembly, the abundance of gene/transcript was calculated in the read count and normalized as FPKM (fragments per kilobase of transcript per million mapped sequence reads). For DEG analysis, statistical analysis was performed by fold change and exact test using edgeR per comparison pair. The significant results were selected on conditions of |FC| ≧ 2 and the exact test p-value < 0.05. If more than one read count value was 0, it was not included in the analysis. For multidimensional scaling analysis, the similarity between samples was graphically shown in a 2D plot using each sample’s normalized value to show the variability of the total data. The quality of produced data was determined by the Phred quality score at each cycle. The numbers of total bases, total reads, mapping efficiencies, GC contents, and the Phred scores (Q20 and Q30) are shown in Table 1.

### Data analysis

Heat maps were depicted by using Heatmapper (http://www.heatmapper.ca/)^32^. The hierarchical clustering analysis was based on Euclidean distance. Each row represents a gene, while each column represents z-scored FPKM of each sample. The green and red gradients represent higher and lower gene expression, respectively. Gene ontology (GO) enrichment analysis was performed in 3 categories such as biological process, cellular component, and molecular function by DAVID Bioinformatics Resources 6.8 (https://david.ncifcrf.gov/)^33^.

### qRT-PCR analysis

Total RNA was extracted using FastGene RNA basic kit (Nippon Genetics). Reverse-transcription was performed by ReverTra Ace qPCR RT Master Mix with gDNA Remover (TOYOBO, Osaka, Japan). Real-time PCR analysis was performed by QuantStudio 3 Real-Time PCR System (ThermoFisher Scientific) using Power SYBR Green PCR Master Mix (ThermoFisher Scientific). The reactions were carried out in triplicate under the following conditions: 10 min at 95 °C, followed by 40 cycles of 15 s at 95 °C and 60 s at 60 °C. All the results were normalized by *Tbp* mRNA levels. The ratio of *Ucp1* to *Fabp4* mRNA was calculated to evaluate tendency of adipocyte browning. The primer sequences specific for *Ucp1* and *Ucp2* mRNA were *Ucp1-specific*: forward 5′-CAAAGTCAAGGCAGACTATGGAC-3′, reverse 5′-TGTTTTTATGATCCAGTCAGCAAG-3′ and *Ucp2-specific*: forward 5′-TCATGGCTGCCTGCACTTC-3′, reverse 5′-AGACAAAGCCAGAGGTGATCAG-3′, respectively. Other primer sequences have been listed previously^22^. The average of three biological replicates was calculated.

### Quantification of mitochondrial DNA

Total genomic DNA was extracted with NucleoSpin Tissue (Takara, Shiga, Japan) from AdMSC cultured with the commercial medium and AdMSC differentiated with SFBAM including either no compound, Ro, or RoFB for 2 weeks. Relative copy numbers of mitochondrial DNA (mtDNA) and nuclear DNA (nuDNA) were measured by qPCR using 10 ng total genomic DNA in each sample and Power SYBR Green PCR Master Mix. mtDNA was normalized to nuDNA in each sample. Primer sequences for quantification of mtDNA and nuDNA were as follows: mtDNA-Fwd, ACACCCTCCTAGCCTTACTAC; mtDNA-Rev, GATATAGGGTCGAAGCCGC; nuDNA-Fwd, AGGGTATCTGGGCTCTGG; NuDNA-Rev, GGCTGAAAAGCTCCCGATTAT^34^.

### Immunoblot analysis

Immunoblot analysis was performed as previously described^21^. Briefly, total proteins were extracted with RIPA buffer [50 mM Tris–HCl (pH 8.0), 0.15 M sodium chloride, 0.5% sodium deoxycholate, 0.1% sodium dodecyl sulphate, 1% NP-40] and both protease and phosphatase inhibitor cocktails (FUJIFILM Wako). The proteins were subjected to 10% SDS-PAGE and transferred to a PVDF membrane (ThermoFisher Scientific). The membranes were blocked with 3% skim milk followed by incubation with UCP1 antibody (MAB6158, R&D Systems, MN, USA), UCP2 antibodies (AF4739, R&D Systems) or β-Actin antibody (A5316, Sigma-Aldrich) at 4 °C overnight. The membranes were incubated with HRP-conjugated secondary antibody (Santa Cruz Biotechnology, CA, USA) for 1 h at room temperature. Immunoreactive bands were detected by Immobilon Western Chemiluminescent HRP Substrate (Merck Millipore, Darmstadt, Germany). Each band intensity was quantified by densitometry using ImageJ software (National Institutes of Health, Bethesda, USA). For evaluation of protein stability, 10 µg/ml Cycloheximide was treated for the time indicated before harvest. Full-length western blots are shown in Supplementary Fig. S7.

### Immunostaining

Immunocytochemistry was performed as previously described^21^. In brief, differentiated and undifferentiated AdMSCs were incubated with either 250 nM MitoTracker Red CMXRos (ThermoFisher Scientific) or 1 µM Lipi-Red (Dojindo, Kumamoto, Japan) for 30 min at 37 °C in 5% CO_2_, according to the manufacturer’s instructions. Then the cells were fixed with 4% paraformaldehyde for 10 min. After washing with PBS, the cells were incubated with PBS containing 0.1% Triton X-100 for 5 min. They were blocked with PBS containing 3% skim milk for 1 h at room temperature. The cells were incubated with UCP1 antibody (ab10983, Abcam, Cambridge, UK) at 1/1000 dilution overnight at 4 °C. After washing with PBS, the cells were further incubated with Alexa Fluor 488 donkey anti-rabbit IgG (ThermoFisher Scientific) for 1 h at room temperature. Cell nuclei were stained with DAPI solution (Dojindo, Kumamoto, Japan). All images were obtained using a BZ-X710-All-in-One Fluorescence Microscope (Keyence, Osaka, Japan). All scale bars represent 100 μm.

### Measurement of OCR

For measurement of OCR by mitochondria, AdMSCs were seeded on a 96-well plate and differentiated by either Ro or RoFB in SFBAM for 2 weeks. As a control, AdMSCs were cultured with the SFBAM only in parallel. These cells were washed and incubated with non-buffered DMEM supplemented with 25 mM glucose, 2 mM Glutamine, and 1 mM pyruvate at 37 °C in a non-CO_2_ incubator 1 h before the measurement. OCR was subsequently measured in the medium by the Seahorse XF96 Extracellular Flux Analyzer (Seahorse Bioscience Inc., MA, USA) according to the manufacturer’s instructions. During the analysis, Oligomycin, FCCP, and Antimycin A/Rotenone were added into each well via an injection apparatus to final concentrations at 2 μM, 0.3 μM, and 0.5 μM, respectively. ECAR was simultaneously measured.

### Statistics

All the results are presented as mean ± SD or SEM. Statistical differences between two groups were evaluated by two-tailed Student’s t-test in the Excel (Microsoft) program. Statistical significance was defined as P-values < 0.05.

## Supplementary Information

Supplementary information.

## Data Availability

The datasets generated during and/or analyzed during the current study are available from the corresponding author on reasonable request.
