# Supplementary material for: Transcriptome analysis reveals brown adipogenic reprogramming in chemical compound-induced brown adipocytes converted from human dermal fibroblasts
Source: Sci Rep. 2021 Mar 3;11:5061. doi: 10.1038/s41598-021-84611-0 (PMC7930091; doi:10.1038/s41598-021-84611-0)
Supplement: Supplementary file 1 — Supplementary information. [file 41598_2021_84611_MOESM1_ESM.pdf]

Transcriptome analysis reveals brown adipogenic reprogramming in chemical compound-induced brown adipocytes converted from human dermal fibroblasts

Yukimasa Takeda[1], Toshikazu Yoshikawa[2], and Ping Dai[1]

[1]Department of Cellular Regenerative Medicine, Graduate School of Medical Science, Kyoto Prefectural University of Medicine, 465 Kajii-cho, Kawaramachi-Hirokoji, Kamigyo-ku, Kyoto 602-8566, Japan; [2]Louis Pasteur Center for Medical Research, 103-5 Tanaka-Monzen-cho, Sakyo-ku, Kyoto 606-8225, Japan

Supplementary Information

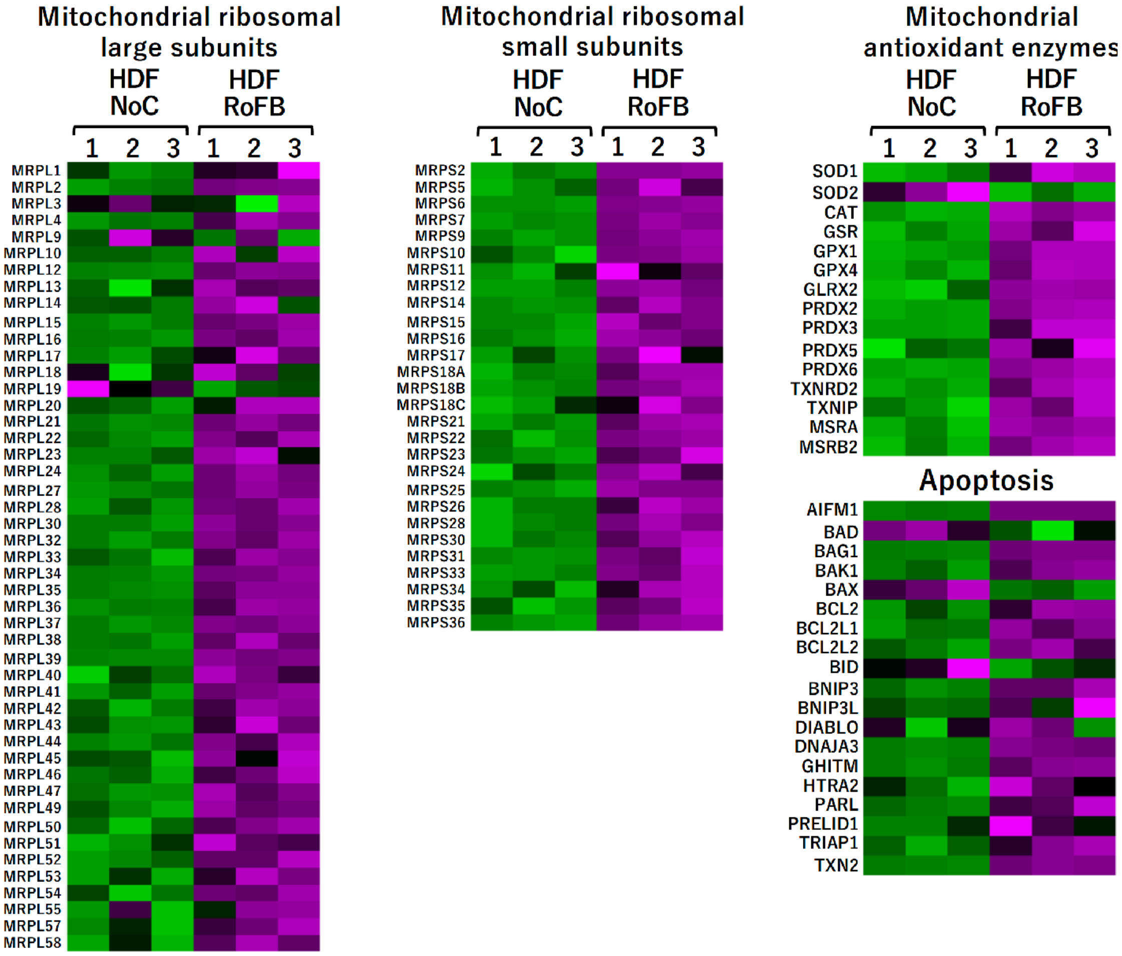

Figure S1. Heat maps represent transcriptional profiles of HDF-NoC and HDF-RoFB in functional groups such as mitochondrial ribosomal large and small subunits, antioxidant enzymes, and apoptosis. The color scale shows z-scored FPKM representing mRNA levels of each gene in green (lower expression) and magenta (higher expression).

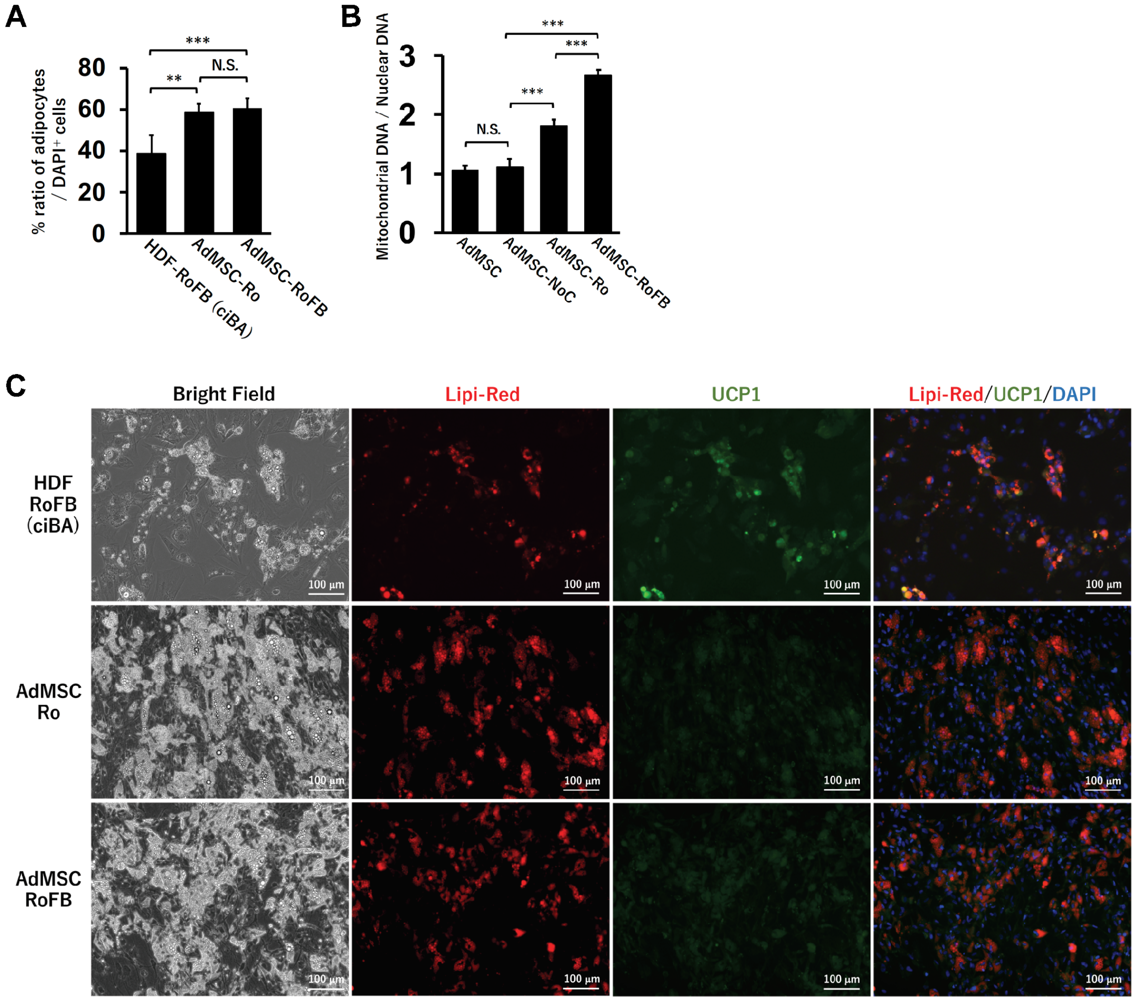

Figure S2. (**A**) To evaluate the differentiation efficiency, the percent ratio of adipocyte-like cells with lipid droplets was calculated. (**B**) Mitochondrial DNA levels were determined by qPCR analysis in the controls and AdMSC-derived adipocytes. The DNA levels were normalized by nuclear DNA. Data represent mean ±SD. Student's t-test: **P<0.01, ***P<0.001, N.S.; not significant. (**C**) Representative images of bright field, lipid staining with Lipi-Red (red), UCP1 protein expression (green), and merged image in ciBAs (HDF-RoFB) and the AdMSC-derived adipocytes (AdMSC-Ro and AdMSC-RoFB). The nuclei were visualized by DAPI (blue). Scale bars represent 100 µm.

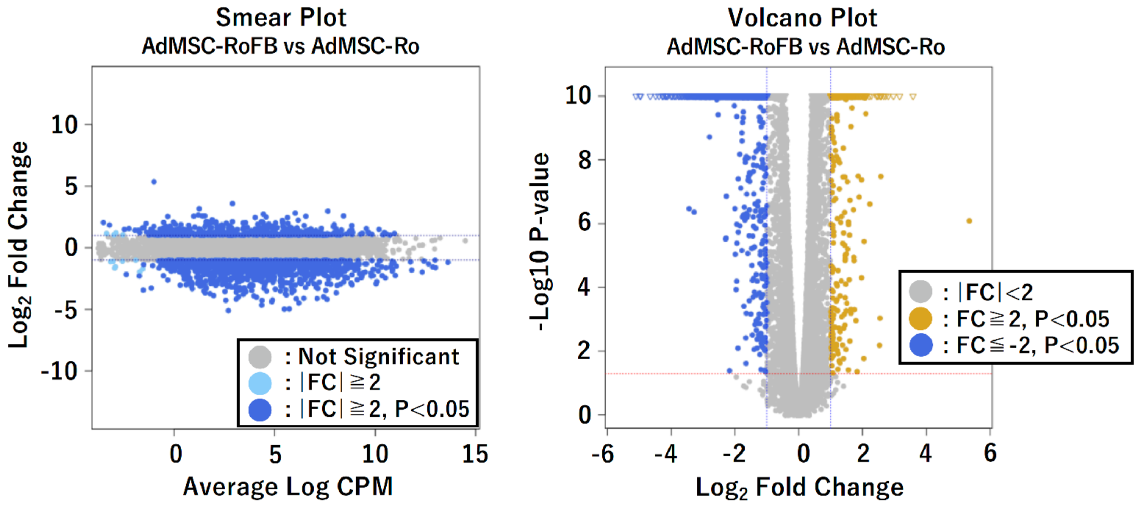

Figure S3. RNA-Seq results in the comparison between AdMSC-Ro and AdMSC-RoFB. Smear and Volcano plots indicate logarithmic fold change, P-value, and CPM (counts per million).

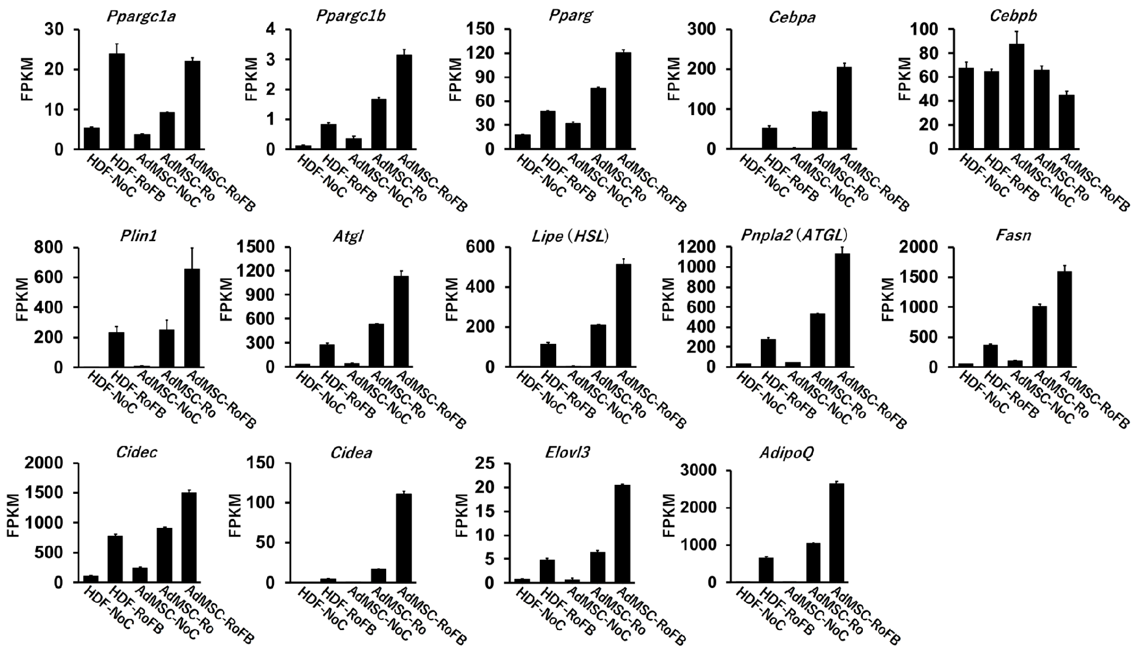

Figure S4. The transcription levels of major adipocyte-enriched genes in ciBA and AdMSC-derived adipocytes. FPKM values were obtained from the RNA-Seq data. Data represent mean ±SD (n=3).

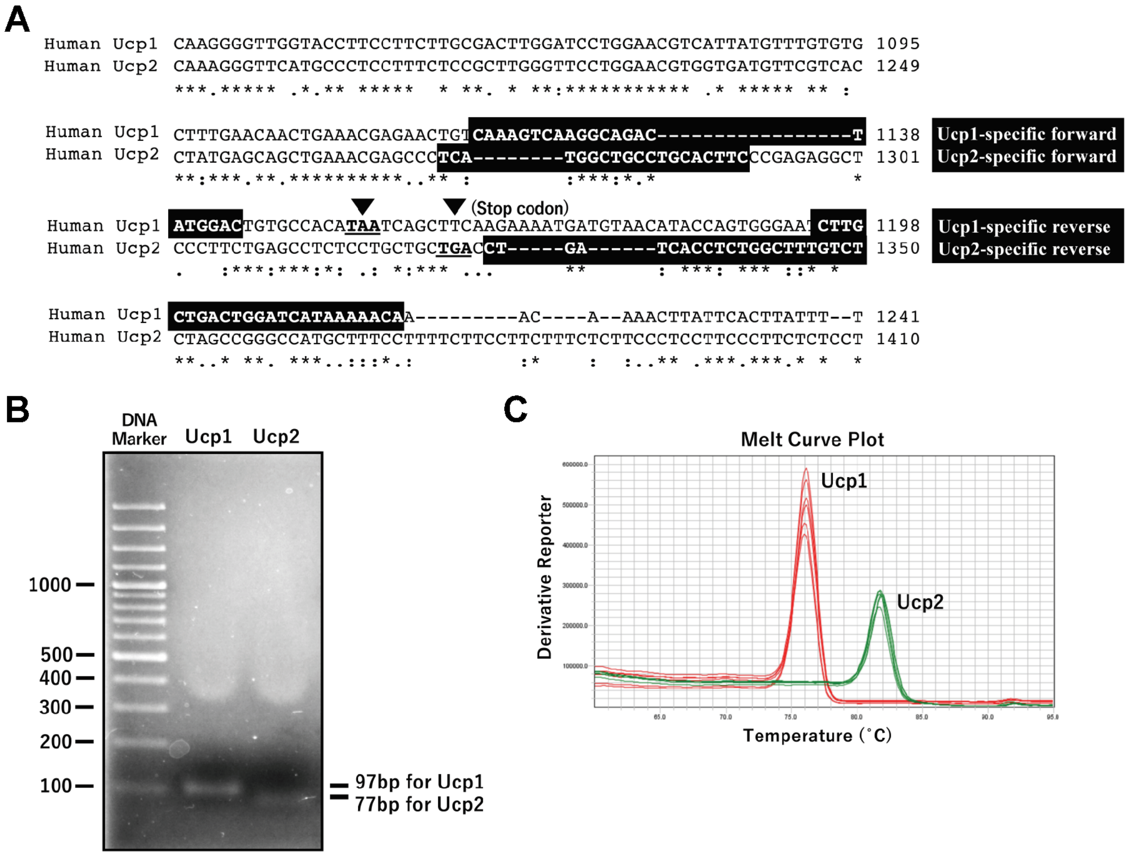

Figure S5. Specificity of PCR primers for human *Ucp1* and *Ucp2*. (**A**) Alignment of partial cDNA sequences of human *Ucp1* and *Ucp2*. The PCR primers highlighted by white letters on a black background were designed around the region with low homology. Arrow heads indicate positions of the stop codon for *Ucp1* and *Ucp2* cDNA. (**B**) A full-length picture of agarose gel electrophoresis shows 97 bp and 77 bp size of PCR products for *Ucp1* and *Ucp2*, respectively. The sequences were confirmed by DNA sequence analysis. (**C**) Single peak of the melting curve plot in real-time PCR reactions represents that the PCR primers yielded a specific PCR product to either *Ucp1* or *Ucp2* without non-specific amplification.

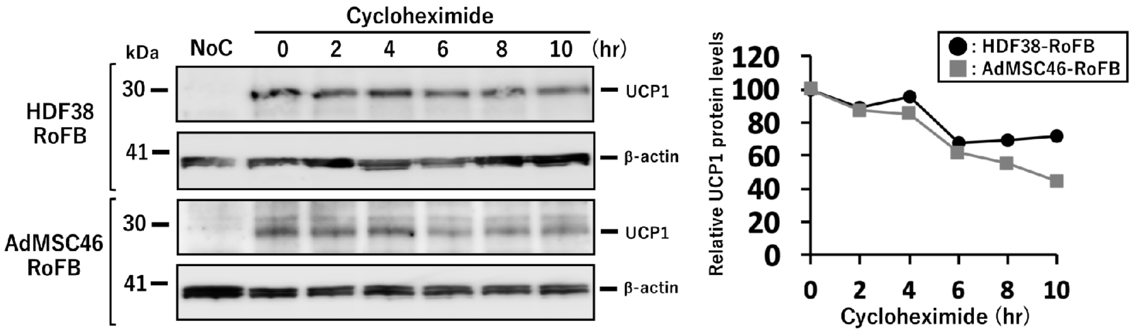

Figure S6. Evaluation of UCP1 protein stability in HDF- and AdMSC-derived adipocytes. UCP1 protein was detected by immunoblotting after treatment with Cycloheximide (10 ug/ml) for the time indicated. The adipocytes were converted by RoFB in SFBAM from HDF38 and AdMSC46. β-Actin protein was a loading control. The band intensities of UCP1 protein were measured by densitometry using ImageJ software and the level at 0 hr was normalized at 100%. Full-length blots are presented in Supplementary Figure S7C.

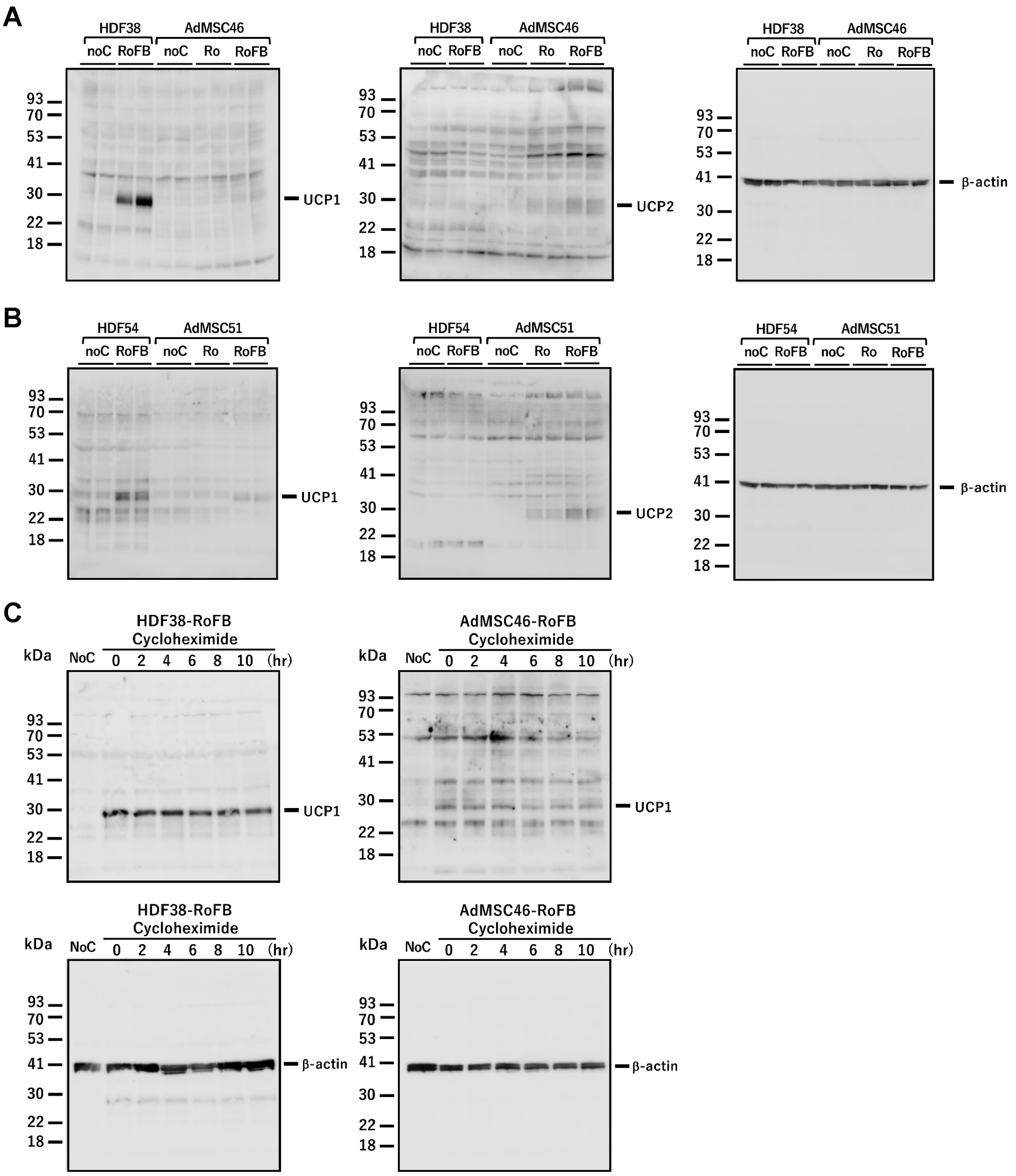

Figure S7. Chemiluminescent images of full-length western blot. (**A-C**) The raw results of western blot analysis are shown for Figure 7A (**A**), Figure 7B (**B**), and Supplementary Figure S6 (**C**).

Table S1. Oxygen consumption rate (pmols/min/10^4 cells) during treatment with no mitochondrial inhibitor, Oligomycin, FCCP, and Antimycin A/Rotenone in AdMSC-derived adipocytes. The proton leak activity was calculated by subtracting OCR during treatment with Antimycin A/Rotenone (corresponding to non-mitochondrial respiration) from OCR during treatment with Oligomycin.

	AdMSC-NoC (Mean OCR ± SEM)	AdMSC-Ro (Mean OCR ± SEM)	AdMSC-RoFB (Mean OCR ± SEM)
No treatment	3.126 ± 0.329	4.042 ± 0.115	3.786 ± 0.371
Oligomycin	1.815 ± 0.229	2.654 ± 0.120	2.952 ± 0.220
FCCP	7.740 ± 0.835	18.11 ± 1.341	18.275 ± 2.212
Antimycin A/ Rotene	1.491 ± 0.175	2.311 ± 0.088	2.228 ± 0.073
Proton Leak Activity	0.324 ± 0.089	0.344 ± 0.052	0.724 ± 0.182

Table S2. Information on human dermal fibroblasts and AdMSCs.

Abbreviation	Lot#	Passage	BMI	Age	Gender	Ethnicity	Site
HDF22	DFM062509	3	29	22	Male	Unknown	Breast
HDF38	DFM090214A	2	23.1	38	Male	Caucasian	Abdomen
HDF54	DDFM052010B	3	21.3	54	Female	African-American	Abdomen
AdMSC38	428Z005.3	2	29.8	38	Male	Caucasian	Abdomen
AdMSC46	394Z027.1	2	Unknown	46	Female	Caucasian	Abdomen
AdMSC51	423Z037.1	2	24	51	Female	Caucasian	Breast
